# Cultural Differences in Opportunity Cost Consideration

**DOI:** 10.3389/fpsyg.2017.00045

**Published:** 2017-01-26

**Authors:** Ning Zhang, Li-Jun Ji, Ye Li

**Affiliations:** ^1^Department of Psychology, Central University of Finance and EconomicsBeijing, China; ^2^Culture and Cognition Lab, Department of Psychology, Queen's UniversityKingston, ON, Canada; ^3^Department of Psychology, Central China Normal UniversityWuhan, China

**Keywords:** cultural differences, opportunity cost consideration, judgment and decision-making

## Abstract

Two studies were conducted to investigate cultural differences in opportunity cost consideration between Chinese and Euro-Canadians. Opportunity cost is defined as the cost of a benefit that must be forgone in order to pursue a better alternative (Becker et al., 1974). In both studies, participants read about hypothetical purchase scenarios, and then decided whether they would buy a certain product. Opportunity cost consideration was measured in two ways: (1) participants' thoughts pertaining to other (nonfocal) products while making decisions; (2) participants' decisions not to buy a focal product (Study 1) or a more expensive product (Study 2). Across both indexes, we found that after controlling for individual difference variables and amount of pocket money, Chinese participants in China considered financial opportunity cost more than Euro-Canadians in Study 1. Similar results were observed in Study 2 when comparing Chinese in Canada with Euro-Canadians However, the cultural effect on opportunity cost consideration was confounded by family income in Study 2. Implications for resource management, limitations of the current research and directions for future research are discussed.

## Introduction

Will you choose to buy a luxury stereo system with your $2000 holiday bonus or spend the money on a wonderful holiday at a Caribbean island resort? Though this may seem to be an easy decision at first glance, you may hesitate before making one choice and forgoing the alternative. Your final decision will depend on how you weigh the opportunity cost of your choice.

Opportunity cost, originally an economics term, is typically defined as “benefits foregone as a result of rejecting the next best alternative action” (Becker et al., 1974, p. 317). With its high relevance to judgment and decision-making, the concept of opportunity cost has drawn substantial attention from researchers in accounting, behavioral economics, and marketing since the 1970s (Neumann and Friedman, 1978; Friedman and Neumann, 1980; Northcraft and Neale, 1986; Frederick et al., 2009).

### Opportunity cost neglect

In classic economic theory, it is assumed that people think and behave rationally to maximize their benefits and minimize their losses; however, research in behavioral economics and psychology has demonstrated that this is not always the case. Instead, irrationality is a common characteristic of human judgments and behavioral decision-making (Tversky and Kahneman, 1974; Thaler, 1980; Ariely, 2008). Rationally speaking, people should consider opportunity cost (i.e., the alternative returns one has to give up) in their decisions to maximize the returns. Yet, empirical evidence has documented that people rarely take opportunity cost into consideration in decision-making, if information about opportunity cost is not explicitly presented (Northcraft and Neale, 1986; Frederick et al., 2009; Shavit et al., 2011). For instance, Northcraft and Neale (1986) found that participants were less likely to consider opportunity cost when opportunity cost information was not mentioned; however, participants altered their decisions to be congruent with the traditional cost/benefit analysis paradigm proposed by economists when they were presented with explicit opportunity cost information. Therefore, opportunity cost information was more likely to be taken into account in decision-making when it was explicitly provided than not. Frederick et al. (2009) recently documented this phenomenon in purchase decision-making. For example, in one study, half of the participants chose between the options “buy this entertainment video” and “not buy this entertainment video” (control condition); the other half of the participants chose between “buy this entertainment video” and “keep the money for other purchases” (opportunity cost salient condition). They found that participants in the control condition were more likely to buy the entertainment video than were participants in the opportunity cost salient condition, suggesting that people were less likely to consider opportunity cost (and therefore purchased the video) when the opportunity cost information was not salient.

#### Factors that influence opportunity cost neglect/consideration

Why do people neglect opportunity costs in making decisions? Researchers from multiple disciplines have attempted to address this question over the past several decades. The explanations include focusing bias in judgment and decision-making (Legrenzi et al., 1993), resource constraints (Spiller, 2011), and individual differences, such as propensity to plan for using money and spending habits (Rick et al., 2008; Spiller, 2011).

#### Focusing bias in judgment and decision-making

One proposed cause of “opportunity cost neglect” is focusing bias in judgment and decision-making. Legrenzi et al. (1993) have shown that people are more likely to focus on the information explicitly presented in reasoning and decision-making tasks. They suggest that focusing on explicit information leads people to neglect alternative options in reasoning and decision-making. Thus, they predicted that providing contextual information should reduce focusing bias and prompt people to consider other choices in decision-making. In support of this prediction, they found that when deciding whether or not to engage in a certain activity in a foreign city (e.g., go to a movie, attend a sporting event), participants in the context group, where information about other options was presented, were more likely to ask questions about alternative options than were participants in the control condition, where information about alternative options was not presented.

Consumer behavior researchers have documented a related phenomenon known as the brand positivity effect (Posavac et al., 2004, 2005), where people evaluate a single brand more favorably if it is presented in isolation rather than with other brand options. In a set of experiments, Posavac et al. (2004, 2005) documented that the brand positivity effect resulted from selective information processing of the focal brand, such that people typically base their evaluation on the information presented about the focal brand without considering information about other brands. In a similar vein, research has demonstrated that subtle differences in framing options as either opportunities (i.e., whether or not to buy a new CD) or choices (i.e., whether to buy a new CD or something else) significantly influence people's preferences (Jones et al., 1998). Specifically, they found that framing options as opportunities led to a higher probability of choosing the target option (i.e., buy the new CD), whereas framing options as choices increased the probability of choosing other alternatives. The results are likely accounted for by the focusing mechanism such that participants focus their attention on the target option in the opportunity framing condition but divide their attention between the target option and other alternative options in the choice framing condition.

#### Resource constraints

Another factor influencing opportunity cost consideration is decision-markers' perceived resource constraints. Specifically, the more resource constraints people perceive, the more likely they will consider opportunity cost in decision-making. In a single lab session, Spiller (2011) simulated a series of 20 purchases, one each weekday for 4 weeks. He assigned participants either to a weekly payment condition where they received $20 store credit each Monday for 4 weeks, resulting in more constraints, or a monthly payment condition, where they received $80 store credit on the first Monday, resulting in less constraint. Participants were instructed to consider the next 3 days' offers before making the purchase decision each day. They were also informed that money spent on 1 day would not be available for future days. Opportunity cost consideration was measured as the proportion of future opportunities considered in making the daily shopping decisions. He found that participants in the weekly payment condition were more likely to consider opportunity cost than were participants in the monthly payment condition. This effect was accounted for by perceived resource constraint.

Zauberman and Lynch (2005) demonstrated a similar pattern such that perceived abundance of resources led people to consider opportunity cost less while committing these resources for future tasks. Participants' perceived abundance of certain resources affected their temporal discounting of future investments of those resources. More specifically, when people perceived that they would have more available time than money in the future, they were more likely to discount future investment of time than future investment of money. Specifically, they made many future time-related commitments that they would not likely accept in the present. The trend was reversed when people perceived that they would have more money than time available in the future. These results suggest that expected abundance of resources lead people to be less likely to consider opportunity cost while committing these resources for future tasks.

#### Individual differences

Research has shown that individual differences also moderate the extent to which people consider opportunity costs (Frederick et al., 2009; Spiller, 2011). For example, in one study, Frederick et al. (2009) found that tightwads, people who do not like to spend money, were more likely to consider opportunity cost and were therefore less influenced by the salience of opportunity cost information than were spendthrifts, people who spend money wastefully. In another study, Spiller (2011) found that, in the absence of resource constraints, planners were more likely to consider opportunity costs than were nonplanners. Accordingly, resource constraints significantly increased the level of opportunity cost consideration among nonplanners but not among planners, as planners were generally more likely to consider opportunity cost already and thus were less susceptible to the resource constraint manipulation. These results highlight the importance of including individual difference variables in research on opportunity cost consideration. In the current research, we explored cultural differences in opportunity cost consideration between Chinese and Euro-Canadians while controlling for individual difference variables such as self-reported habit of opportunity cost consideration (OCC; Spiller, 2011), propensity to plan for money (PPM; Lynch et al., 2010), and difficulty of spending or controlling spending (STS; Rick et al., 2008), in order to see whether cultural differences exist beyond these individual difference variables. Although, individual difference in opportunity cost consideration is conceptually related to our main dependent variables (e.g., presence of opportunity cost thoughts and choice), we decided to control for it in the current research, as previous research suggests that cultural differences observed at the group level are not necessarily reducible to individual differences (Na et al., 2010).

#### Culture and opportunity cost consideration

Most of the research on opportunity cost consideration has been conducted in North America with participants of European descent (Northcraft and Neale, 1986; Vera-Muñoz, 1998; Frederick et al., 2009; Spiller, 2011), therefore, little is known about whether opportunity cost consideration varies across cultures. Based on previous cultural research, particularly research considering cultural differences in context sensitivity (Nisbett et al., 2001; Nisbett and Norenzayan, 2002; Nisbett, 2003; Heine, 2010), we predicted that East Asians would consider opportunity cost more than would European North Americans.

Previous research in culture and cognition has demonstrated that East Asians are generally more sensitive and attentive to context than are North Americans (Ji et al., 2000; Masuda and Nisbett, 2001; Nisbett et al., 2001; Nisbett and Norenzayan, 2002; Nisbett, 2003; Masuda et al., 2008). For example, Ji et al. (2000) found that, compared to European Americans, East Asians were more likely to detect covariation between events and were more field dependent when making perceptual judgments. Similarly, Masuda and Nisbett (2001) found that, compared with Americans, Japanese recalled more information about contexts and relationships, and were more likely to recognize an object when it was presented in its original setting instead of a novel setting.

In everyday life, people frequently face decisions that involve choosing one option among multiple alternatives. The nonfocal alternatives can be considered as the context for decision-making. In most situations, however, alternative options are not explicitly presented. Given that East Asians are more sensitive to contexts than European North Americans, would they also be more readily attentive to alternative options and therefore consider opportunity cost more in purchase decision-making? The present research aims to address this question by comparing hypothetical purchase decisions of Chinese and Euro-Canadians.

Based on previous research on opportunity cost consideration and on research considering cultural differences in sensitivity to context, we hypothesized that Euro-Canadians would consider opportunity cost less than would Chinese. Furthermore, we predicted that making opportunity cost information salient would significantly increase European Canadian participants' likelihood of considering opportunity cost, compared to those in the control condition, whereas the salience of opportunity cost information would have a weaker or no effect on Chinese participants' opportunity cost consideration. We conducted two studies to test these hypotheses.

## Study 1

The purpose of Study 1 was to examine whether culture and the salience of opportunity cost information would influence opportunity cost consideration in a purchase decision. Study 1 also measured individual difference variables, including habits of considering opportunity cost, propensity to plan for money, and spending habits, as previous research has shown that these variables are relevant to opportunity cost consideration (Frederick et al., 2009; Spiller, 2011).

### Methods

#### Participants

One hundred and twenty-one European Canadian students recruited from the subject pool at a Canadian University (100 women, 21 men; *M*_*age*_ = 18.17 years, *SD* = 0.96) and 119 Chinese university students recruited through in-class announcement at a Chinese University (86 women, 33 men; *M*_*age*_ = 19.51 years, *SD* = 0.86) participated in Study 1. European Canadian participants received course credit for their participation; Chinese participants received small gifts (a ballpoint pen) as a compensation for their participation.

#### Procedure

Participants read a decision-making scenario adapted from Frederick et al. (2009). In the scenario, participants imagined that they had been saving money to make some purchases. While visiting a shopping mall, they came across a special sale on a new travel backpack that cost $29.99, which happened to be their favorite style. Participants were randomly assigned to either the control condition or opportunity cost salient condition. Participants in the control condition read two options: “Buy this backpack” (option A) or “Not buy this backpack” (option B). Participants in the opportunity cost salient condition read: “Buy this backpack” (option A) or “Keep the $29.99 for other purchases” (option B). Thus, the only difference between the two conditions was how option B was phrased. By highlighting the possibility of other potential purchases, the wording in the opportunity cost salient condition emphasized the opportunity costs of buying the backpack. Following Frederick et al. (2009), choosing not to buy the backpack was considered as a decision consistent with opportunity cost consideration. The study materials were created in English, and then translated to Chinese by three bilingual researchers using the back translation method suggested by Brislin (1970) to ensure their equivalence across cultures. Following conventions used in behavioral economics, the price of the backpack ($29.99) was converted to Chinese Yuan (¥110) based on the Big Mac Index (2012).

For the main dependent variables, participants indicated their likelihood of choosing option A or B on an 8-point bipolar scale (1 = *definitely choose option A*, 8 = *definitely choose option B*). A larger number in choice indicates a higher level of opportunity cost consideration. They also listed any thoughts they had while deciding which option to choose. The order in which participants rated their choices and listed their thoughts was counterbalanced among participants^1^. Next, participants indicated the amount of money they imagined to have saved and how their choice would influence their ability to buy other things on a 7-point scale (1 = *not at all*, 7 = *a great deal*)^2^.

Next, we measured individual differences in participants' spending styles on some rating scales. Heine et al. (2002) have warned us of the danger of comparing results of rating scales across cultures due to the reference group effect, thus we only included these scales to control for individual differences instead of comparing them across cultures. Participants firstly completed the three-item Opportunity Cost Consideration Scale (OCC; Spiller, 2011) on a 7-point scale (1 = *Strongly disagree*, 7 = *Strongly agree*), which measures an individual's general tendency to consider opportunity cost in making purchase decisions. A sample item is “I often think about the fact that spending money on one purchase now means not spending money on some other purchase later.” Then, they finished the six-item Propensity to Plan for Money-Long Term Scale (PPM; Lynch et al., 2010) on a 7-point scale (1 = *Strongly disagree*, 7 = *Strongly agree*), which assesses an individual's propensity to make plans for spending money in the long term. A sample item is “I set financial goals for the next 1–2 years for what I want to achieve with my money.” Afterwards, they completed the four-item Spendthrift-Tightwad Scale (STS; Rick et al., 2008) which measures the extent to which individuals experience pain while spending money, with higher numbers indicating a spendthrift (or less pain of spending money). The STS scale includes four scenario questions. For example, “Some people have trouble limiting their spending: they often spend money—for example on clothes, meals, vacations, phone calls—when they would do better not to. Other people have trouble spending money. Perhaps because spending money makes them anxious, they often don't spend money on things they should spend it on. How well does the first description fit you? That is, do you have trouble limiting your spending? (1 = *never*, 5 = *always*).”

Finally, participants reported demographic information, including age, gender, ethnicity, amount of pocket money they typically had each month, and family income^3^. All the studies were approved by the Queen's General Research Ethics Board.

### Results

#### Individual difference measures

Following previous research, responses to the Opportunity Cost Consideration Scale (unstandardized Cronbach α = 0.82, and 0.76 for Euro-Canadians and Chinese, respectively) and the Propensity to Plan for Money Scale (unstandardized Cronbach α = 0.90, and 0.87 for Euro-Canadians and Chinese, respectively) were averaged, respectively, and responses to the STS (unstandardized Cronbach α = 0.78, and 0.67 for Euro-Canadians and Chinese, respectively) were summed up as an index of being a spendthrift (i.e., people with low pain of spending money). The self-reported individual difference variables indicated that Euro-Canadians (*M* = 5.01, *SD* = 1.32) reported a stronger habit of considering opportunity cost than did Chinese (*M* = 3.98, *SD* = 1.48), *t*_(238)_ = 5.74, *p* < 0.001; Euro-Canadians (*M* = 4.43, *SD* = 1.44) also reported a significantly higher propensity to plan for money than did Chinese (*M* = 3.22, *SD* = 1.25), *t*_(238)_ = 6.97, *p* < 0.001. Chinese (*M* = 15.92, *SD* = 3.61) scored higher on the Spendthrift and Tightwads Scale than did Euro-Canadians (*M* = 14.59, *SD* = 4.58), *t*_(1, 235)_ = 2.48, *p* = 0.014, indicated that they experienced a lower level of pain in spending money.

Although Euro-Canadians reporting a stronger habit of considering opportunity cost than Chinese did not seem to support our hypothesis, correlation analyses indicated that this self-reported tendency of considering opportunity cost was not significantly correlated with either of the opportunity cost consideration indexes (choice or presence of opportunity cost thoughts) for Chinese and only significantly correlated with the presence of opportunity cost thoughts for Euro-Canadians (See Table 1 for correlations among variables). As Euro-Canadians and Chinese differ in self-reported tendency of considering opportunity cost (OCC scale), propensity to plan for money (PPM scale), and pain of spending (STS scale) and that these individual difference variables were not consistently correlated with the indexes of opportunity cost consideration, we controlled all the individual difference variables and pocket money in the main analyses to explore whether cultural differences in opportunity cost consideration would exist beyond individual differences.

**Table 1:** **Means, standard deviations, and correlations of measured variables (Study 1)**.

**Variables**	**Mean**	**SD**	**1**	**2**	**3**	**4**
**EURO-CANADIANS**						
1. OCC	5.01	1.32				
2. PPM	4.43	1.44	0.405^**^			
3. STS	14.59	4.58	−0.466^**^	−0.416^**^		
4. Choice	2.58	1.76	0.089	0.112	−0.247^**^	
5. OC thoughts	0.24	0.43	0.262^**^	0.156	−0.211^*^	0.394^**^
**CHINESE**						
1. OCC	3.98	1.45				
2. PPM	3.22	1.25	0.280^**^			
3. STS	15.92	3.61	−0.238^**^	−0.242^**^		
4. Choice	2.88	1.71	0.168	0.170	−0.323^**^	
5. OC thoughts	0.30	0.46	0.166	0.128	−0.018	0.243^**^

* *p < 0.05*,

** *p < 0.01*.

*As OC thought is a categorical variable, Spearman rank correlation coefficients are reported for OC thoughts*.

#### Presence of thoughts on opportunity cost

Blind to the hypothesis, Two English-Chinese bilingual research assistants coded the thoughts that participants listed in terms of whether or not participants mentioned opportunity cost (e.g., other items they planned to buy) in making the decision. The inter-coder agreement on thoughts was 94% for Euro-Canadian, and 92% for Chinese. Disagreements between coders were resolved through discussion.

After effect coding the two categorical variables—culture (−1 = Euro-Canadians, 1 = Chinese) and condition (−1 = control, 1 = opportunity cost salient), we first conducted a binary logistic regression analysis with culture, condition, and the interaction between culture and condition as independent variable and presence of opportunity cost thoughts (1 = present, 0 = absent) as the dependent variable. Only condition was a significant predictor of the presence of opportunity cost thoughts, *B* = 0.39, *Wald* (1) = 6.66, *p* = 0.01, *Exp*(B) = 1.47. That is, participants in the Opportunity Cost Salient condition were 1.47 times more likely than those in the control condition to think about opportunity costs while making the purchase decision. The effect of culture, *B* = 0.16, *Wald* (1) = 1.08, *p* = 0.30, and the interaction between culture and condition, *B* = 0.03, *Wald* (1) = 0.044, *p* = 0.83, were not significant.

Next, we conducted a similar binary logistic regression, but adding all the individual difference measures, and amount of pocket money as covariates. As differences in amount of pocket money may influence people's likelihood of considering opportunity cost, we controlled for the amount of pocket money (after converting Chinese yuan into Canadian dollars based on the exchange rate at the time of data collection and standardizing it within each culture). The results revealed a main effect of condition, *B* = 0.45, *Wald*(1) = 7.60, *p* = 0.006, *Exp*(B) = 1.56, and a main effect of culture, *B* = 0.54, *Wald*(1) = 7.11, *p* = 0.008, *Exp*(B) = 1.72. Specifically, participants in the Opportunity Cost Salient condition were 1.56 times more likely that those in the control condition to think about opportunity costs while making the purchase decision; Chinese were 1.72 times more likely than Euro-Canadians to think about opportunity costs while making the purchase decision. The results also revealed a significant effect of OCC average, *B* = 0.36, *Wald*(1) = 6.77, *p* = 0.009, *Exp*(B) = 1.43. Thus, while controlling for the other variables, the greater tendency participants reported themselves considering opportunity cost, the more likely they would generate opportunity cost thoughts. No other effect approached significance, *Wald*s < 2.06, *ps* > 0.15 (see Table 2 for coefficients and standard errors of the predictors included in the models)^4^.

**Table 2:** **Results of logistic regression for study 1 (DV = presence of OC thoughts)**.

	**Predictors**	***B***	***S.E***.	**Wald**	***df***	***p***	**Exp(B)**
Model 1 (without controlling individual difference variables	Condition	0.387	0.150	6.657	1	0.01	1.473
	Culture	0.156	0.150	1.082	1	0.298	1.169
	Condition by Culture	0.032	0.150	0.044	1	0.833	1.032
	Constant	−1.023	0.150	46.486	1	0.000	0.36
Model 2 (controlling all individual difference variables and pocket money)	Condition	0.447	0.162	7.602	1	0.006	1.564
	Culture	0.541	0.203	7.11	1	0.008	1.717
	Condition by Culture	0.104	0.160	0.42	1	0.516	1.11
	Pocket money	−0.406	0.267	2.314	1	0.128	0.666
	OCC average	0.359	0.138	6.767	1	0.009	1.432
	PPM average	0.175	0.130	1.815	1	0.178	1.192
	TS total	0.031	0.046	0.465	1	0.495	1.032
	Constant	−3.081	1.232	9.918	1	0.002	0.021
Model 3 (similar to analysis 2, without controlling OCC average)	Condition	0.407	0.157	6.697	1	0.01	1.503
	Culture	0.441	0.196	6.058	1	0.025	1.554
	Condition by Culture	0.059	0.156	0.144	1	0.704	1.061
	Pocket money	−0.471	0.282	2.79	1	0.095	0.624
	PPM average	0.252	0.126	3.987	1	0.046	1.287
	TS total	−0.002	0.043	0.002	1	0.969	0.998
	Constant	−2.016	0.959	4.422	1	0.035	0.133

#### Choice

After effect coding the two categorical variables—culture (−1 = Euro-Canadians, 1 = Chinese) and condition (−1 = control, 1 = opportunity cost salient), we first conducted a regression analysis to explore the effects of culture, condition, and the interaction between culture and condition on choice without covariates. The overall model was marginally significant, *F*_(3, 235)_ = 2.46, *R*^2^ = 0.03, *p* = 0.064. Consistent with the results based on presence of opportunity cost thoughts, only condition was a significant predictor of choice, *B* = 0.27, *t*_(235)_ = 2.40, *p* = 0.017. The effect of culture, *B* = 0.11, *t*_(235)_ = 0.94, *p* = 0.35, and the interaction between condition and culture, *B* = −0.09, *t*_(235)_ = −0.79, *p* = 0.43, were not significant.

Next, we conducted a linear regression analysis while controlling for the individual difference variables and the amount of pocket money. The overall model was significant, *F*_(7, 219)_ = 3.65, *R*^2^ = 0.105, *p* = 0.001. The results revealed that condition, *B* = 0.29, *t*_(219)_ = 2.51, *p* = 0.013, and STS, *B* = −0.09, *t*_(219)_ = −2.96, *p* = 0.003, were significant predictors of choice. These results indicated that participants in the opportunity cost salient condition were less likely than participants in the control condition to buy the backpack; the less pain one reported in paying, the more likely they would choose to buy the backpack. Culture was a marginally significant predictor of decision, *B* = 0.24, *t*_(219)_ = 1.75, *p* = 0.081, indicating a trend for Chinese to be less likely than Euro-Canadians to buy the backpack. No other effects approached significance, −0.54 < *ts* < 0.82, *ps* > 0.41 (see Table 3 for coefficients and standard errors of the predictors included in the model)^5^.

**Table 3:** **Results of linear regression for study 1(DV = Choice)**.

	**Predictors**	***B***	***S.E***.	***B***	***T***	***p***	**95% CI**
Model 1 (without controlling individual difference variables	Condition	0.272	0.113	0.154	2.403	0.017	[0.049, 0.496]
	Culture	0.106	0.113	0.06	0.936	0.35	[−0.117, 0.329]
	Condition by Culture	−0.089	0.113	−0.051	−0.788	0.431	[−0.313, 0.134]
	Constant	2.694	0.113		23.769	0.001	[2.47, 2.917]
Model 2 (controlling all individual difference variables and pocket money)	Condition	0.287	0.115	0.161	2.508	0.013	[0.062, 0.513]
	Culture	0.242	0.138	0.136	1.751	0.081	[−0.03, 0.515]
	Condition by Culture	−0.062	0.115	−0.035	−0.538	0.591	[−0.288, 0.164]
	Pocket money	−0.068	0.132	−0.038	−0.516	0.606	[−0.329, 0.192]
	OCC average	0.025	0.092	0.021	0.273	0.785	[−0.156, 0.206]
	PPM average	0.076	0.093	0.063	0.812	0.418	[−0.108, 0.259]
	TS total	−0.095	0.032	−0.225	−2.959	0.003	[−0.159, −0.032]
	Constant	3.768	0.826		4.558	0.001	[2.138, 5.395]
Model 3 (similar to analysis 2, without controlling OCC average)	Condition	0.286	0.114	0.160	2.504	0.013	[0.061, 0.511]
	Culture	0.234	0.135	0.131	1.735	0.084	[−0.032, 0.501]
	Condition by Culture	−0.065	0.114	−0.036	−0.569	0.57	[−0.289, 0.16]
	Pocket money	−0.068	0.132	−0.038	0.512	0.609	[−0.328, 0.192]
	PPM average	0.082	0.090	0.068	0.911	0.363	[−0.095, 0.259]
	TS total	−0.098	0.031	−0.231	−3.158	0.002	[−0.169, −0.037]
	Constant	3.892	0.685		5.678	0.000	[2.541, 5.243]

### Discussion

In Study 1, participants generally considered opportunity cost to a greater extent in the opportunity cost salient condition than in the control condition, indexed by participants' likelihood of reporting opportunity cost thoughts while making the purchase decision and by their choice of not to buy the backpack. When individual difference variables and the amount of pocket money were controlled for, Chinese were in general more likely than Euro-Canadians to report opportunity cost thoughts and to make choices consistent with opportunity cost consideration.

Study 1 had several limitations. First, the price of the backpack ($29.99/¥110) seemed too low, which led to a floor effect such that there was a high level of intention to buy the backpack and therefore little intention not to buy it. Indeed, a majority of the participants' (105 Euro-Canadians and 101 Chinese) responses to the 8-point bipolar scale were below or equal to four (i.e., “slightly likely to buy the backpack”). Strong preference for buying the backpack could suppress participants' likelihood of considering opportunity cost in making the choices. Second, as the decision involved whether or not to buy the backpack (instead of choosing between two products), participants who did not need a backpack (or were not interested in getting one) were more likely to choose not to buy it regardless of whether they considered opportunity cost or not. Lastly, although we intended to make the price equivalent by using the Big Mac index to convert Canadian dollars to Chinese Yuan, it was still possible that $29.99 to Euro-Canadians was not equivalent to 110 Yuan to Chinese, which may confound the results.

## Study 2

Study 2 was conducted to test the same predictions as in Study 1 with the following improvements. First, we introduced a more expensive product for university students—a laptop, which should help to prevent the floor effect observed in Study 1. Second, rather than deciding whether or not to buy a product such as in Study 1, participants in Study 2 had to choose between two similar products. This should render participants' need for or interest in the product less relevant in their decisions. Third, we recruited both Chinese and Canadian participants at a Canadian university and presented the product price in Canadian dollars. This excluded the possibility that price difference and product information might confound participants' responses. Study 2 also explored whether the condition effect observed in Study 1 would be replicated with a different way of manipulating the salience of opportunity cost information—opportunity cost priming through a purchase-listing task.

### Methods

#### Participants

Seventy-five European Canadian students (61 women, 14 men; *M*_*age*_ = 18.29 years, *SD* = 0.71), and 63 Chinese students (46 women, 17 men; *M*_*age*_ = 20.03 years, *SD* = 2.66) at a Canadian University participated in Study 2^6^. Participants either received course credit or monetary compensation for their participation. All study materials were presented in English with product prices in Canadian dollars.

#### Procedure

Adapting a design from Frederick et al. (2009), we randomly assigned participants to the opportunity cost priming condition or the control condition. All participants completed a decision-making task, in which they had to decide whether to buy a laptop with a 500 GB hard drive or a similar laptop that was $100 cheaper with a 320 GB hard drive. The only difference between the priming and control conditions was that participants in the priming condition completed a seemingly unrelated purchase-listing task prior to the decision-making task, whereas participants in the control condition were not given this purchase-listing task. In the purchase-listing task, participants listed, on a blank page of paper, things (either one item or multiple items) that they could buy with $100. The purchase-listing task was meant to prime participants with the opportunity costs of spending an additional $100 for the more expensive laptop.

Participants then read the laptop scenario and indicated their choice on a scale from 1 to 8, with 1 meaning they would definitely choose the more expensive laptop, and 8 meaning that they would definitely choose the cheaper laptop. Thus, a higher number in choice indicated a higher level of opportunity cost consideration. Next, they listed any thoughts they had while deciding which option to choose and indicated to what extent making the purchase would influence their ability to buy other things (1 = *not at all*, 7 = *a great deal*). Afterwards, participants completed the 3-item Opportunity Cost Consideration Scale (Spiller, 2011). Due to time constraints, we only had time to include one individual difference measure. Finally, participants answered demographic questions such as age, gender, ethnicity, family income, average amount of pocket money for each month, and perceived financial security^7^.

### Results

#### Individual difference measure

The Cronbach's alpha for the Opportunity Cost Consideration (OCC) scale is 0.86 and 0.80 for Euro-Canadians and East Asians, respectively. Unlike in Study 1, Euro-Canadians (*M* = 5.24, *SD* = 1.29) and Chinese (*M* = 4.91, *SD* = 1.32) did not differ from each other on OCC, *t*_(136)_ = 1.41, *p* = 0.14. Thus, cross-cultural differences in the OCC scale did not appear reliable. Besides, Euro-Canadians (*M* = 4.25, *SD* = 1.60) and Chinese (*M* = 4.41, *SD* = 1.44) did not differ in how making the purchase would influence their ability to buy other things, *t*_(136)_ = −0.61, *p* = 0.54. Euro-Canadians (*M* = 5.17, *SD* = 1.30) and Chinese (*M* = 4.87, *SD* = 1.28) also did not significantly differ from each other on perceived financial security, *t*_(136)_ = 1.36, *p* = 0.18. They also did not significantly differ from each other on amount of pocket money (*M* = 204.27, *SD* = 221.34 for Euro-Canadians, vs. *M* = 267.75, *SD* = 385.64 for Chinese), *t*_(133)_ = −1.20, *p* = 0.23. However, Euro-Canadians (*M* = 4.89, *SD* = 1.37) reported a higher level of family income than Chinese (*M* = 3.43, *SD* = 1.71), *t*_(132)_ = 5.51, *p* < 0.001.

As seen in Table 4, self-reported tendency of considering opportunity cost was not significantly correlated with either presence of opportunity cost thought (*r* = −0.052) and their likelihood of choosing the cheaper laptop (*r* = 0.012) among Euro-Canadians. Among Chinese, self-reported tendency of considering opportunity cost was not correlated with the presence of opportunity cost thoughts (*r* = 0.011), but negatively correlated with choice (*r* = −0.276, *p* = 0.05), indicating that those Chinese who reported a higher level of opportunity cost consideration were less likely to buy the cheaper laptop, which was puzzling, but also indicating an inconsistency between the rating scale score and people's choices.

**Table 4:** **Means, standard deviations, and correlations of measured variables (Study 2)**.

**Variables**	**Mean**	**SD**	**1**	**2**
**EURO-CANADIANS**				
OCC	5.24	1.29		
Choice	4.41	2.31	0.012	
OC thoughts	0.12	0.33	−0.052	0.306^**^
**CHINESE**				
OCC	4.91	1.32		
Choice	5.37	2.20	−0.276^*^	
OC thoughts	0.29	0.46	0.011	0.444^**^

* *p < 0.05*,

** *p < 0.01*.

*As OC thought is a categorical variable, Spearman rank correlation coefficients are reported here for OC thoughts*.

#### Presence of thoughts considering opportunity cost

Two research assistants, blind to the research hypothesis, coded participants' thoughts using the same scheme as in Study 1. The inter-coder agreement was 86%; disagreements were resolved through discussion. After effect coding for the two categorical variables—Culture (−1 = Euro-Canadians, 1 = Chinese) and Condition (−1 = control, 1 = opportunity cost priming), a binary logistic regression was conducted to examine how participants' opportunity cost thought (0 = not present, 1 = present) could be predicted by culture, condition, and the interaction between culture and condition. Only culture was a significant predictor of the presence of opportunity cost thoughts, *B* = 0.53, *Wald* (1) = 5.43, *p* = 0.02, *Exp*(B) = 1.70. That is, Chinese in Canada were 1.70 times more likely than Euro-Canadians to think about opportunity costs while making the purchase decision. The effect of condition, *B* = 0.20, *Wald* (1) = 0.78, *p* = 0.38, and the interaction between culture and condition, *B* = 0.18, *Wald* (1) = 0.63, *p* = 0.43, were not significant.

Next, we conducted another binary logistic regression analysis with effect coded culture, condition, and the interaction between culture and condition as predictor, while controlling for individual differences in OCC, family income, pocket money, and perceived financial security. The results revealed a significant effect of family income on the presence of opportunity cost thoughts, *B* = −0.48, *Wald* (1) = 7.63, *p* = 0.006, *Exp*(B) = 0.618; a marginally significant effect of pocket money on the presence of opportunity cost thoughts, *B* = −0.002, *Wald* (1) = 2.73, *p* = 0.098, *Exp*(B) = 0.998. The effect of culture was not significant, *B* = 0.25, *Wald* (1) = 0.89, *p* = 0.35, *Exp*(B) = 1.29. None of the other effects reached significance, *Wald* (1)s < 2.69, *ps* > 0.10. This indicates that with every one unit increase in their level of family income, people will be 1.62 times less likely to think about opportunity cost while making the purchase decision (see Table 5 for coefficients and standard errors of the predictors included in the model)^8^.

**Table 5:** **Results of logistic regression for study 2 (DV = presence of OC thoughts)**.

	**Predictors**	***B***	***S.E***.	**Wald**	***df***	***p***	**Exp(B)**
Model 1 (without controlling individual difference variables	Condition	0.201	0.229	0.778	1	0.378	1.223
	Culture	0.533	0.229	5.428	1	0.02	1.703
	Condition by Culture	0.181	0.229	0.628	1	0.428	1.199
	Constant	−1.462	0.229	40.905	1	0.001	0.232
Model 2 (controlling OCC average and financial variables)	Condition	0.415	0.253	2.681	1	0.102	1.514
	Culture	0.254	0.269	0.888	1	0.346	1.289
	Condition by Culture	0.289	0.251	1.327	1	0.249	1.336
	OCC average	−0.218	0.19	1.315	1	0.251	0.804
	Family income	−0.481	0.174	7.629	1	0.006	0.618
	Pocket money	−0.002	0.001	2.734	1	0.098	0.998
	Financial security	0.382	0.212	2.407	1	0.121	1.388
	Constant	0.251	1.443	0.03	1	0.862	1.285
Model 3 (similar to analysis 2, without controlling OCC average)	Condition	0.365	0.247	2.191	1	0.139	1.44
	Culture	0.279	0.267	1.092	1	0.296	1.322
	Condition by Culture	0.28	0.249	1.262	1	0.261	1.323
	Family income	−0.473	0.173	7.478	1	0.006	0.623
	Pocket money	−0.002	0.001	2.113	1	0.146	0.998
	Financial security	0.346	0.209	2.741	1	0.098	1.413
	Constant	−1.003	0.944	1.129	1	0.288	0.367

To further understand cultural differences in the presence of opportunity cost thoughts when making purchase decisions, we explored whether Chinese were more likely than Euro-Canadians to report opportunity cost thoughts even when they were both choosing the cheaper option. Among all participants who chose the cheaper option, Chinese participants (40.9%) were marginally more likely than Euro-Canadians (21.6%) to report opportunity cost thoughts, $\chi_{\left(1,\text{N}=81\right)}^{2}$ = 3.43, *p* = 0.06. This implies that opportunity cost thoughts were more accessible among Chinese than Euro-Canadians when they decided to buy the cheaper option.

#### Choice

After effect coding for the two categorical variables—Culture (−1 = Euro-Canadians, 1 = Chinese) and Condition (−1 = control, 1 = opportunity cost priming), we firstly conducted a linear regression analysis to explore the effect of culture, condition, and the interaction between culture and condition in predicting people's decisions. The overall model was significant, *F*_(3, 134)_ = 2.83, *R*^2^ = 0.06, *p* = 0.041. The results revealed that culture was the only significant predictor of decision, *B* = 0.48, *t*_(134)_ = −2.48, *p* = 0.014, such that Chinese were more likely than Euro-Canadians to buy the cheaper laptop.

Next, we conducted another linear regression analysis while controlling for OCC, family income, pocket money, and perceived financial security^9^. The overall model was significant, *F*_(7, 123)_ = 3.26, *R*^2^ = 0.16, *p* = 0.003. The results revealed that family income was a significant predictor of decision, *B* = −0.46, *t*_(123)_ = −3.24, *p* = 0.002, such that those reported a higher level of family income were less likely to buy the cheaper laptop. The results also revealed that OCC was a marginally significant predictor of decision, *B* = −0.29, *t*_(123)_ = −1.88, *p* = 0.063, such that those reported a higher likelihood of considering opportunity cost were less likely to make an opportunity-cost consistent decision. This suggests a discrepancy in people's perception of their tendency and what they would likely do when making purchase decisions. The effect of culture was not significant, *B* = 0.14, *t*_(123)_ = 0.62, *p* = 0.53 (see Table 6 for coefficients and standard errors of the predictors included in the model)^10^.

**Table 6:** **Results of linear regression for study 2 (DV = Choice)**.

	**Predictors**	***B***	***S.E***.	***b***	***t***	***p***	**95% CI**
Model 1 (without controlling individual difference variables	Condition	−0.052	0.193	−0.023	0.272	0.786	[−0.434, 0.329]
	Culture	0.479	0.193	0.208	2.480	0.014	[0.097, 0.861]
	Condition by Culture	−0.296	0.193	−0.129	−1.533	0.128	[−0.678, 0.086]
	Constant	4.87	0.193		25.218	0.000	[4.488, 5.251]
Model 2 (controlling OCC average and financial variables)	Condition	0.087	0.198	0.037	0.438	0.662	[−0.305, 0.478]
	Culture	0.138	0.221	0.059	0.623	0.534	[−0.30, 0.575]
	Condition by Culture	−0.249	0.196	−0.107	−1.274	0.205	[−0.636, 0.138]
	OCC average	−0.288	0.153	−0.164	−0.188	0.063	[−0.592, 0.015]
	Family income	−0.456	0.141	−0.233	−3.243	0.002	[−0.735, −0.178]
	Pocket money	−0.001	0.001	−0.141	−1.585	0.116	[−0.002,0.000]
	Financial security	0.203	0.168	0.114	1.205	0.231	[−0.13, 0.536]
	Constant	7.443	1.193		6.23	0.000	[5.071, 9.795]
Model 3 (similar to analysis 2, without controlling OCC average)	Condition	0.032	0.198	0.014	0.163	0.871	[−0.359, 0.423]
	Culture	0.184	0.222	0.079	0.830	0.408	[−0.255, 0.623]
	Condition by Culture	−0.248	0.198	−0.107	−1.256	0.211	[−0.639, 0.143]
	Family income	−0.447	0.142	−0.327	−3.148	0.002	[−0.728, −0.166]
	Pocket money	−0.001	0.001	−0.105	−1.194	0.235	[−0.002, 0.001]
	Financial security	0.222	0.170	0.125	1.308	0.193	[−0.114, 0.558]
	Constant	5.779	0.814		7.101	0.000	[4.168, 7.389]

### Discussion

In Study 2, we found a higher degree of opportunity cost consideration among Chinese than among Euro-Canadians, but this effect was no longer significant when OCC, family income, pocket money, and perceived financial security were included in the analysis. Indeed, family income confounded the effect of culture on decision. Overall, compared to Euro-Canadians, Chinese participants reported a lower level of family income, suggesting they may be more constrained in resource. However, there was no cultural difference in responses to the question, “how would making this purchase influence your ability of buying other things?” This is also inconsistent with the “cushion hypothesis” proposed by Hsee and Weber (1999) such that Chinese would be more financially risk-taking than Euro-Canadians because they would be more likely to receive financial help from their family. Indeed, other than family income, none of the other resource related questions (e.g., pocket money, perceived financial security, perceived ability of buying other things) modified or confounded the cultural effect on opportunity cost consideration. Given that culture and family income were negatively correlated in this study (Spearman *r* = −0.44, *p* = 0.01), it was difficult to separate the two completely. Future study should try to disentangle the two factors.

In the present study, opportunity cost priming did not have any significant effect on participants' purchase decisions, which did not replicate Frederick et al. (2009). The only differences between the present study and Study 4 in Frederick et al. (2009) were that the price difference between the two products changed from $20 to $100 and that the product changed from a cellphone to a laptop. It is possible that other factors unexplored in the current study moderated the effect of opportunity cost priming on people's purchase decisions.

## General discussion

Across two studies, we explored whether opportunity cost consideration varies across cultures. Our first hypothesis was that Euro-Canadians would be less likely to consider opportunity cost than Chinese. Study 1 revealed that, while controlling for individual differences in habits of considering opportunity cost or propensity to plan for money, Chinese were more likely than Euro-Canadians to think about opportunity cost and to make decisions consistent with opportunity cost consideration. However, in Study 2, the cultural differences in opportunity cost consideration disappeared when family income was included as a covariate. We discussed the limitations of the study in the limitation section.

The second hypothesis, that Euro-Canadians would benefit more than Chinese from the salience of opportunity cost information, was not supported. In Study 1, the results indicated that participants from both culture groups benefited equally from the opportunity cost salience manipulation. In Study 2, salience of opportunity cost information did not have a significant effect on either culture group. It is unclear to what extent other factors unexamined in the present research, such as the particular product used in each study, might have influenced the impact of the opportunity cost salience manipulation. Future research should investigate these other factors.

In Study 2, family income confounded the effect of culture on choice, however, it is not clear how family income accounted for the cultural difference in opportunity cost consideration. One possibility is that cultural differences in family income may lead to different levels of perceived resource constraint. However, the two cultural groups did not differ in perceived affordability to buy other things, financial security, or average amount of pocket money in Study 2, and none of these factors explained the relationship between family income and choice. Another possibility is that family income may have afforded people with different family environments and life experiences, which could foster different attitudes toward spending money and therefore influence their purchasing behaviors. For example, lower family income may foster a stronger intention of planning for spending money or a stronger habit of comparing different products before making a purchase decision. As indicated in Study 1, however, propensity to plan for spending money, based on self-report, was actually higher among (rich) Euro-Canadians than among (less rich) Chinese. Furthermore, greater planning propensity among Euro-Canadians did not lead to greater opportunity cost consideration, in either thoughts or decisions. Thus, future research is warranted to disentangle the effects of culture and family income on opportunity cost consideration.

The current research contributes to research on culture and decision-making. Previous research on opportunity cost consideration was predominantly conducted with North American participants (Becker et al., 1974; Northcraft and Neale, 1986; Frederick et al., 2009; Spiller, 2011). To the best of our knowledge, the current research is the first to explore cultural differences in opportunity cost consideration. The current results suggest that opportunity cost consideration varies, at least to some extent, across cultures. This research echoes Weber and her colleagues' advocate on the importance of taking culture into account in understanding many decision-making phenomena (Weber and Hsee, 2000; Weber and Morris, 2010). Future research is required to further explore the issue cross-culturally and to investigate the underlying mechanisms.

The present study also highlights the importance of using multiple indices in cross-cultural research on judgment and decision-making. In the current research, results based on participants' self-report on the opportunity cost consideration scale were inconsistent across two studies. Self-reported opportunity cost consideration was not in line with the other two indices (i.e., choice rating and presence of opportunity cost thoughts) of opportunity cost consideration either. For example, Chinese participants' intention to choose the cheaper laptop was negatively correlated with their self-reported level of opportunity cost consideration in Study 2 (*r* = −0.28, *p* = 0.05), which was puzzling. Future research is required to develop and verify the validity of these measures in general and for cross-cultural research in particular.

### Limitations

One limitation of the present research is that factors not measured in the current research may have attenuated the effect of priming opportunity cost in decision-making. The priming method affected opportunity cost consideration among American participants in Frederick et al. (2009), but no such effect was observed with Canadian participants in our Study 2, although Euro-Canadians' mean responses were trending in the expected direction. One possibility is that Frederick et al. (2009) had a larger sample than we did. Another possibility is that buying a laptop is a relatively large purchase for university students, and the size of the purchase could moderate the effect of priming on opportunity cost consideration. Besides, due to the fact that both Euro-Canadians and Chinese participants were recruited from Queen's University in Study 2, this could make it harder for us to find the cultural differences in opportunity cost consideration as demonstrated in Study 1 as undergraduate students attending the same university may be more similar to each other rather than different. Future research could explore the moderating effect of these factors across a more culturally diverse samples.

Second, choosing whether or not to buy a product (or the likelihood of choosing to buy a product) might not be an optimal index of opportunity cost consideration because the decision is hypothetical, and it is likely influenced by multiple factors such as price, need, and preference. This problem is especially true in the cross-cultural context, as previous research has demonstrated substantial cross-cultural differences in the determinants of consumer behavior (Luna and Gupta, 2001; de Mooij and Hofstede, 2011). In the current studies, fortunately, we also elicited participants' thoughts during decision making, in addition to their ratings of choice. These thoughts provide additional insights to the phenomenon being studied. For instance, the presence of opportunity cost thoughts in Study 2 indicated that Chinese were slightly more likely to think about opportunity costs than were Euro-Canadians even when they made similar decisions (e.g., choosing the cheaper laptop). This finding shows that the choice intention measure, used in previous research (Frederick et al., 2009) and the current research, may not be the best indication of opportunity cost consideration. Future research is warranted to further explore cultural differences in opportunity cost consideration in real purchase decision-making contexts.

Furthermore, measurement invariance tests suggested that the individual difference variables included in the current studies (i.e., self-reported opportunity cost consideration, propensity to plan for money, and experience of pain in spending money) only reached configural invariance but not for metric or scalar invariance across cultures^11^.

This limitation precludes meaningful cross-cultural comparisons on these individual differences. The inconsistencies in the correlations between the self-reported individual difference measures and the target dependent variables (e.g., presence of opportunity cost thoughts, choice) also suggest problems that make it not valid to compare them cross-culturally.

### Future directions

The results of Study 2 revealed that family income difference confounded the relationship between culture and opportunity cost consideration. However, it is not clear how family income led to opportunity cost consideration in the current study and whether there are psychological variables (other than the socioeconomic variables) responsible for cultural differences in opportunity cost consideration. Thus, future research is warranted to further investigate the effects of culture and family income on opportunity cost consideration. For instance, future research could compare Euro-Canadians and Chinese with similar family incomes to explore whether the culture main effect observed in the current study could be replicated. If there are indeed cultural differences in opportunity cost consideration after matching socioeconomic variables (e.g., family income), a fruitful next step would be to investigate the underlying mechanisms of this phenomenon, such as holistic thinking or sensitivity to context. Although we speculated that sensitivity to the decision context was one potential factor to influence cultural differences in people's likelihood of considering opportunity cost, we did not manipulate or measure sensitivity to decision context in the current research. Future research is worthwhile to explore the role of cultural differences in decision contexts sensitivity in accounting for cultural differences in opportunity cost consideration.

While the current research focuses on opportunity cost involving money, future research can expand it to other types of opportunity costs, such as time, relationships, and environmental impact. Exploring cultural differences in opportunity cost consideration in these nonfinancial domains not only has the advantage of circumventing the interfering effect of culturally contingent socioeconomic indices (e.g., family income) encountered in the current research, but will also enrich our understanding of cultural effects on decision making and resource management in general.

## Ethics statement

Joan Stevenson, Ph.D. Professor and Chair, General Research Ethics Board, Queen's University. Participants were provided with a hard copy or online version of the consent form when came to the lab to participate in the studies. The study only began after they sign the consent form and they have freedom to withdraw from the study at any time.

## Author contributions

NZ designed the study under the supervision of LJ. NZ collected the data in Canada, YL collected the data in China. NZ analyzed the data and drafted the manuscript under the supervision of LJ. LJ provided critical revisions for the manuscript.

## Funding

The research was supported by a grant from the Social Science and Humanities Research Council of Canada (SSHRCC Grant 435-2012-1279).

### Conflict of interest statement

The authors declare that the research was conducted in the absence of any commercial or financial relationships that could be construed as a potential conflict of interest.

## Footnotes

^1^Data analysis showed no order effect; therefore, it will not be discussed further.

^2^Chinese (*M* = 4.08, *SD* = 1.70) and Euro-Canadians (*M* = 4.18, *SD* = 1.49) did not differ on this measure, *t*_(229)_ = 0.48, *p* = 0.63.

^3^Participants reported the amount of pocket money they have per month in Canadian dollars (for Euro-Canadians, *M* = 166.84, *SD* = 164.13) or in Chinese Yuan (for Chinese, *M* = 429.38, *SD* = 387.88). Family income was reported in Canadian dollars on a 6-point scale (1 = less than $25,000, 6 = more than $125,000) for Euro-Canadians and Chinese Yuan on an 11- point scale (1 = less than ¥30,000, 11 = more than ¥1,000,000) for Chinese, therefore, they cannot be compared directly. Within Euro-Canadians, family income was not correlated with their choice, *r* = −0.12, *p* = 0.19, but marginally correlated with the presence of opportunity cost thoughts, *r* = −0.16, *p* = 0.09; the amount of pocket money was marginally correlated with their choice, *r* = −0.17, *p* = 0.07 and significantly correlated with the presence of opportunity cost thoughts, *r* = −0.20, *p* = 0.03. Within Chinese, family income was not correlated with their choice (*r* = −0.13, *p* = 0.17) nor with presence of opportunity cost thoughts (*r* = −0.04, *p* = 0.64); amount of pocket money was not correlated with their choice, *r* = −0.11, *p* = 0.25, but marginally correlated with presence of opportunity cost thoughts (*r* = −0.17, *p* = 0.07).

^4^Including OCC (scale) average as a covariate in the model or not produced a similar pattern of results.

^5^Including OCC (scale) average as a covariate in the model or not produced a similar pattern of results.

^6^Only 24 Chinese participants answered the question regarding how long they had lived in North America (Canada/US). The average was 12.02 years (*SD* = 5.81).

^7^There were no significant differences between Euro-Canadians and Chinese in their perceived affordability to buy other things, in their self-reported financial security, and in the amount of pocket money they had each month, *ts* <1.37, *ps* > 0.17. The median family income for Euro-Canadians was in the range of $100,000–$125,000 and the median family income for Chinese was in the range of $50,000–$75,000.

^8^For those curious readers, we conducted a similar binary logistic regression analysis as in Study 1, with OCC and pocket money as covariates. We found that only culture was a significant predictor of the presence of opportunity cost thoughts, *B* = 0.52, *Wald* (1) = 5.06, *p* = 0.024, *Exp*(B) = 1.70. None of the other effects reach significance, *Walds* (1) <1.82, *ps* > 0.17. This indicated that, while controlling for OCC and pocket money, Chinese in Canada were 1.70 times more likely to think about opportunity cost while making the purchase decision. A separate analysis with presence of opportunity cost thoughts as the dependent variable, culture, condition, and the interaction between culture and condition as the independent variables, only controlling family income, pocket money, and perceived financial security revealed similar pattern of results as that when individual differences in opportunity cost consideration (OCC average) was also controlled in the model. Including OCC (scale) average as a covariate in the model or not produced a similar pattern of results.

^9^We did another linear regression on decision, with culture and condition, and the interaction between culture and condition as predictors, and controlling for OCC and pocket money. The overall model was significant, *F*_(5, 129)_ = 2.48, *R*^2^ = 0.09, *p* = 0.035. The results revealed a significant effect of culture, *B* = −0.48, *t*_(129)_ = −2.41, *p* = 0.017, and a marginally significant effect of OCC, *B* = −0.27, *t*_(129)_ = −1.73, *p* = 0.086. None of the other effects reached significance, −1.54 < *t*s(129) < −0.18, *ps* > 0.12. Therefore, while controlling for OCC and pocket money only, Chinese in Canada were more likely to buy the cheaper option than Euro-Canadians.

^10^Including OCC (scale) average as a covariate in the model or not produced a similar pattern of results.

^11^We conducted further analyses using LISREL to test measurement invariance of the individual difference measures included in Study 1 and 2. The results revealed that the Opportunity Cost Consideration Scale (OCC) used in Study 1 only reached configural invariance [$\chi_{\left(0\right)}^{2}$ = 0, *p* = 1.00, RMSEA = 0.000 for the unconstrained model; $\chi_{\left(2\right)}^{2}$ = 9.44, *p* = 0.0089, RMSEA = 0.177 for the constrained model; $\Delta \chi_{\left(2\right)}^{2}$ = 9.44, *p* < 0.01]; the Propensity to Plan for Money scale did not reach configural invariance [$\chi_{\left(18\right)}^{2}$ = 128.08, *p* < 0.001, RMSEA = 0.227 for the unconstrained model; $\chi_{\left(23\right)}^{2}$ = 146.38, *p* < 0.001, RMSEA = 0.212 for the constrained model; $\Delta \chi_{\left(5\right)}^{2}$ = 18.30, *p* = < 0.01]; the Spendthrift-Tightwad Scale (STS) reached configural invariance [$\chi_{\left(5\right)}^{2}$ = 6.80, *p* = 0.24, RMSEA = 0.055 for the unconstrained model; $\chi_{\left(8\right)}^{2}$ = 14.12, *p* = 0.0788, RMSEA = 0.08 for the constrained model; $\Delta \chi_{\left(3\right)}^{2}$ = 7.32, *p* > 0.05], but the mode for metric invariance was not significantly better than the model for configural invariance; the Opportunity Cost Consideration Scale (OCC) used in Study2 reached configural invariance [$\chi_{\left(0\right)}^{2}$ = 0, *p* = 1.00, RMSEA = 0.000 for the unconstrained model; $\chi_{\left(2\right)}^{2}$ = 2.81, *p* = 0.25, RMSEA = 0.077 for the constrained model; $\Delta \chi_{\left(2\right)}^{2}$ = 2.81, *p* > 0.05], but the mode for metric invariance was not significantly better than the model for configural invariance. Therefore, the individual difference variables did not reach metric or scalar invariance for cross-cultural comparison in the current research.
